# Caffeic Acid Phenethyl Ester Ameliorates Calcification by Inhibiting Activation of the AKT/NF-κB/NLRP3 Inflammasome Pathway in Human Aortic Valve Interstitial Cells

**DOI:** 10.3389/fphar.2020.00826

**Published:** 2020-07-07

**Authors:** Ming Liu, Fei Li, Yuming Huang, Tingwen Zhou, Si Chen, Geng Li, Jiawei Shi, Nianguo Dong, Kang Xu

**Affiliations:** Department of Cardiovascular Surgery, Union Hospital, Tongji Medical College, Huazhong University of Science and Technology, Wuhan, China

**Keywords:** human aortic valve disease, natural product, polyphenolic compound, NF-κB pathway, inflammasome

## Abstract

Calcific aortic valve disease (CAVD) occurs *via* a pathophysiological process that includes inflammation-induced osteoblastic differentiation of aortic valvular interstitial cells (AVICs). Here, we investigated the role of the anti-inflammatory compound caffeic acid phenethyl ester (CAPE) in inhibiting CAVD. Human AVICs were isolated and cultured in osteogenic induction medium (OM) with or without 10 μM CAPE. Cell viability was assessed using CCK8 assays and calcified transformation of AVICs was evaluated by Alizarin Red staining and osteogenic gene/protein expression. RNA-sequencing was conducted to identify differentially expressed genes (DEGs) and enrichment in associated pathways, as potential molecular targets through which CAPE inhibits osteogenic induction. The regulatory effects of CAPE on activation of the AKT/NF-κB and NLRP3 inflammasome were evaluated by Western blot analysis and immunofluorescent staining. CAPE slowed the growth of AVICs cultured in OM but did not show significant cytotoxicity. In addition, CAPE markedly suppressed calcified nodule formation and decreased gene/protein expression of RUNX2 and ALP in AVICs. Gene expression profiles of OM-induced AVICs cultured with or without CAPE revealed 518 common DEGs, which were highly enriched in the NOD-like receptor, PI3K-AKT, and NF-κB signaling pathways. Furthermore, CAPE inhibited phosphorylation of AKT, ERK1/2, and NF-κB, and suppressed NLRP3 inflammasome activation in AVICs cultured in OM. Thus, CAPE is implicated as a potent natural product for the prevention of CAVD by inhibiting activation of the AKT/NF-κB pathway and NLRP3 inflammasome.

**Graphical Abstract:**
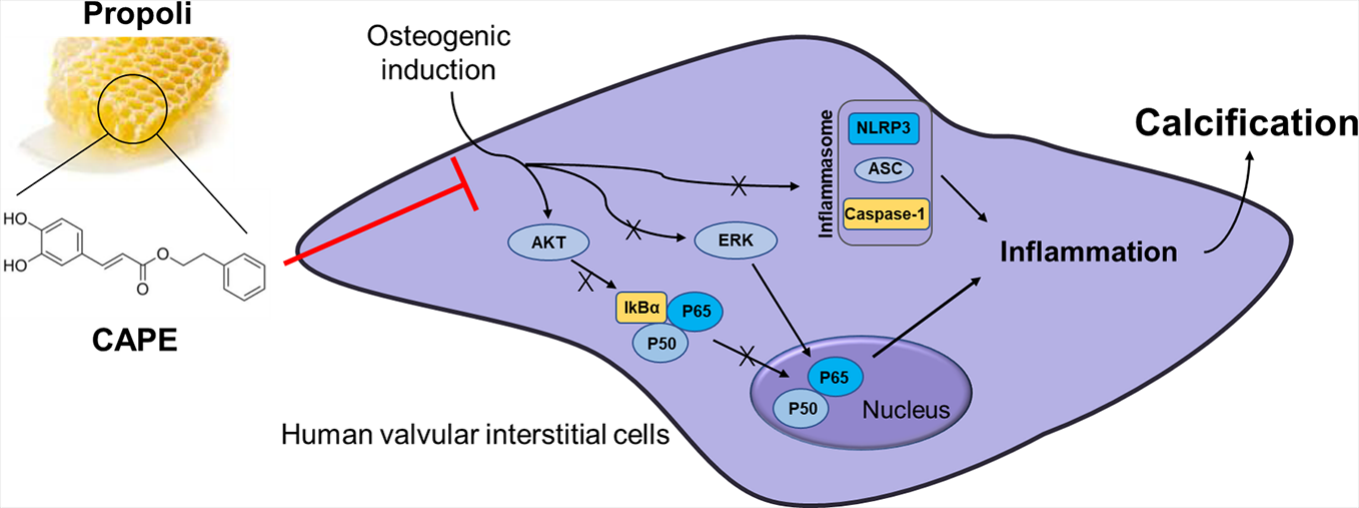
CAPE significantly inhibits OM-induced calcification and phenotypic transformation of AVICs *via* signaling pathways including AKT, ERK1/2, NF-κB/NLRP3 inflammasome.

## Introduction

Calcified aortic valve disease (CAVD), the most common Cardiac valvular disease worldwide, is characterized by valvular calcification, leading to aortic stenosis and subsequent heart failure (Nkomo et al., 2006). Increasing evidence obtained over the past decade suggests that CAVD is not simply a passive degenerative process, but an active pathological condition similar to that associated with atherosclerosis, including processes such as lipoprotein deposition, chronic inﬂammation, and osteoblastic differentiation of aortic valve interstitial cells (AVICs) (Li et al., 2013; P et al., 2014; Rutkovskiy et al., 2017). Currently, there is no effective pharmacological therapy for CAVD other than surgical or interventional aortic valve replacement (Da et al., 2015).

Both *in vitro* and clinical studies have suggested that a sequence of active osteogenic processes contribute to CAVD, and that osteogenic activity is initiated by inflammation (Nadra et al., 2005; Marincheva-Savcheva et al., 2011; New and Aikawa, 2011; Pawade et al., 2015). AVICs are the principle cell type found within aortic valve leaflets and participate in the process of CAVD primarily by inducing both inflammation and osteoblastic differentiation (Rutkovskiy et al., 2017). This inﬂammatory damage is a critical factor that causes CAVD. Therefore, the search for effective treatment modalities for valvular calcification, such as the use of medication to regulate inflammatory responses, has important clinical value and significance, and may effectively delay the onset of aortic valve calcification.

Caffeic acid phenethyl ester (CAPE), a natural polyphenolic compound, is mainly found in the bark of conifer trees, but is also present in propolis from honeybee hives (Wu et al., 2011). Previous studies have shown that CAPE is effective against various pathologies such as infections, oxidative stress, inflammation, cancer, diabetes, neurodegeneration, and anxiety (Parlakpinar et al., 2005; Celik and Erdogan, 2008; Tolba et al., 2016; Nie et al., 2017). Moreover, CAPE has been demonstrated to inhibit NF-κB and to contribute to anti-inflammatory processes (Celik and Erdogan, 2008; Nie et al., 2017). In our previous studies, we confirmed that inflammatory responses accelerate the formation of valvular calcification (Xu et al., 2018; Huang et al., 2019; Xu et al., 2019a). Therefore, we investigated the anti-calcification effect of CAPE.

In this study, we found that CAPE significantly inhibited osteogenic medium (OM)-induced calcification in human AVICs. To further clarify the mechanism by which CAPE inhibits AVIC calcification, we conducted high-throughput RNA-sequencing quantification to analyze global changes in gene expression induced in AVICs cultured in OM with or without CAPE. Finally, we confirmed the involvement of inhibition of the AKT/NF-κB signaling pathway and NLRP3 inflammasome in the mechanism by which CAPE inhibits AVIC calcification.

## Materials and Methods

### Cell Culture and Treatments

This human study was approved by the ethics committee of the Union Hospital, Tongji Medical College, Huazhong University of Science and Technology (China). Human specimens were obtained from the Department of Cardiovascular Surgery, Union Hospital, Tongji Medical College, Huazhong University of Science and Technology. All participants provided written informed consent according to the Declaration of Helsinki. From October 2018 to April 2019, aortic valve leaﬂets were obtained intra-operatively from patients (**Table 1**) undergoing the Bentall operation due to acute type I aortic dissection. Patients with a history of infective endocarditis, rheumatic heart disease, or a genetic syndrome were excluded. The degree of calcification of the aortic valve samples was determined as previously described (Li et al., 2017). Briefly, isolated leaflets were digested in medium containing 1 mg/mL collagenase I at 37°C for 30 min. After vortexing, the leaflets were further digested with a fresh solution of 1 mg/mL collagenase medium at 37°C for 8–10 h. After repeated aspiration to break up the tissue mass, the suspension was centrifuged at 300 ×*g* for 10 min. Subsequently, the cells were resuspended and cultured in M199 growth medium, supplemented with 100 U/mL penicillin, 100μg/mL streptomycin and 10% fetal bovine serum at 37°C under 5% CO_2_. Cells were used in experiments at passage 3. For the osteogenic differentiation model, hVICs were cultured in osteogenic induction medium (OM) (Cyagen Biosciences, HUXMA-90021) to stimulate osteogenic differentiation according to previously described protocols (Huang et al., 2020; Zhou et al., 2020). CAPE was purchased from Selleck (Cat. No. S7414) and dissolved in DMSO to yield a 10 mM stock solution. The treatment groups were as follows: Control group (without OM and CAPE), OM-treated group (OM alone) and OM + CAPE-treated group.

**Table 1:** Sample information.

Sample type	Degree	Sex	Age
Health	0	Male	36
Health	0	Male	53
Health	0	Female	42

### Cell Viability Analysis

The cells were seeded on the 48-well plates at the cell density of 5 × 10³ cells/well and cultured in 10% FBS-DMEM medium for 24 h. Subsequently, the medium was changed into serum-free medium for starvation for 12 h. Then, the cells were treated with different final concentrations of CAPE (0-25 μM) for 72 h, and IC50 was calculated. In addition, the cells were also treated with 10 μM CAPE for 5 days. Cell viability in the experiments was detected with a CCK-8 assay (Bimake, Houston, TX). Briefly, at the end of each time interval, cell samples were washed with PBS and incubated with serum free medium containing 10% CCK-8 reagent. After 3 h of incubation at 37°C under 5% CO2, aliquots were pipetted into a 96-well plates and measured at 490 nm using an enzyme labeling instrument.

### Calcification Analysis

Cells were seeded into 12-well plates and cultured for 2–3 days to reach confluence. Cells were then cultured in either OM with or without 10 μM CAPE for 21 days. The degree of cell calcification was measured by Alizarin Red S (Sciencell, 0223) staining according to the manufacturer’s instructions. In brief, after 21 days of treatment, the cells were fixed with 4% paraformaldehyde (PFA) and then incubated with 2% Alizarin Red S solution for 30 min at room temperature. After washing twice with distilled water, images were captured for evaluation of the degree of calcification. For quantitative analysis, cells were incubated in a 10% aqueous solution of cetylpyridinium chloride and the amount of Alizarin Red S dye released from the extracellular matrix was quantified by spectrophotometry at a wavelength of 550 nm.

### qRT-PCR Assay

Cells were harvested using a Trizol reagent (Invitrogen, Carlsbad, CA), followed by RNA isolation. Each sample cDNA was reverse transcribed using the Revert Aid First Strand cDNA Synthesis Kit (Thermo Fisher Scientific, Waltham, MA). Then, the reverse transcription product was used as a template to perform real-time polymerase chain reaction (PCR) on a Step One Plus thermal cycler (Applied Biosystems, Foster City, CA) using a PowerUp™ SYBR™ Green Master Mix (Applied Biosystems) following the manufacturer’s guide. All the primers were referenced from the previous study, and synthesized by Invitrogen; primer sequences are shown in **Supplementary Table 1**. The final data were analyzed by the 2-ΔΔct method.

### Western Blot Analysis

After culture for 48 h or 7 days, cells were harvested, lysed in the RIPA buffer containing protease and further bored. The protein samples were resolved by SDS-PAGE (4%–20% gels) and then transferred to PVDF membranes using a wet-transfer system. After blocking with 5% (wt/vol) skimmed milk in TBS-T solution (50 mM Tris/HCL, pH 7.6, 150 mM NaCl and 0.1% (vol/vol)Tween-20) at room temperature for 1 h, membranes were incubated at 4°C overnight with primary detection antibodies for RUNX2 (CST, 8486s), ALP (Zenbio, 220678), GAPDH (Proteintech, 60004-1-Ig), AKT (CST, 4685s), p-AKT (CST, 9614), IκBα (CST, 4814s), p-IκBα (CST, 2859s), p-ERK (Zenbio, 310065), ERK (Zenbio, 340373), NLRP3 (CST, D4D8T), ASC (CST, E1E3I), and P20 (ag-0042). The membranes were then incubated for 1 h at room temperature with the appropriate horseradish peroxidase (HRP)-conjugated secondary detection antibodies diluted in 5% (wt/vol) skimmed milk in TBS-T solution. Finally, the immunoreactive bands were developed using SuperSignal West Femto Maximum Sensitivity Substrate (Thermo Fisher Scientific), and the images were analyzed using Image J software.

### Detection of mRNA Profiles

RNA-sequencing (RNA-seq) quantification was utilized to investigate changes in cell mRNA profiles among the different treatments performed. Cells were harvested using a Trizol reagent (Invitrogen, Carlsbad, CA), followed by RNA isolation. Isolated RNA was sent to BGI Co., LTD (Wuhan, China) for RNA-seq performed on BGISEQ-500. Sequencing results were further analyzed using the “R (version 3.5.1)” to identify differential expression genes (DEGs) and then a Kyoto Encyclopedia of Genes and Genomes (KEGG) pathway enrichment analysis was performed.

### Cell Immunostaining Assays

The AVICs were cultured with different treatments for 48 h. The cell immuno-staining was performed according to the previous protocols. The primary antibodies RUNX2 (Abcam, ab23981), ALP (Zenbio, 220678), and P65 (Cell Signaling Technology: 8242) were used. After secondary antibody incubation, the cell nucleus was stained with DAPI (Roche) for 15 min, then the samples were observed and captured by fluorescent microscopy (Zeiss).

### Statistical Analysis

All data were expressed as the mean ± standard deviation (SD). All semiquantitative measurements were captured using Image J software. Differences between groups were evaluated by analysis of variance (ANOVA). *P*-values less than (<) 0.05 were considered to indicate statistical significance.

## Results

### Effect of CAPE on Cell Viability and Morphology

**Figure 1:**
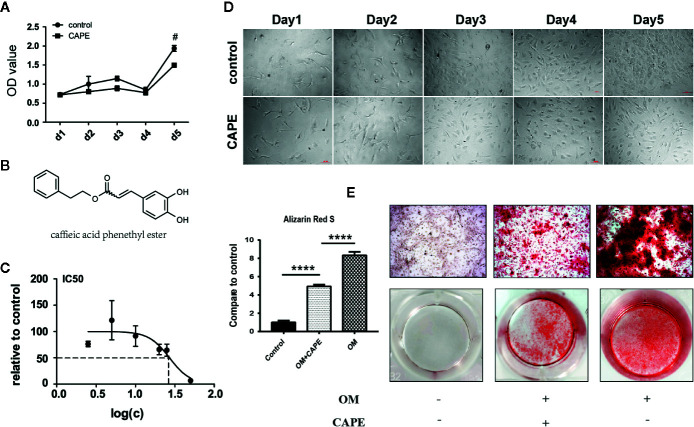
Viability and calcification of AVICs with treatment of CAPE. **(A)** Cell proliferation curve with or without CAPE treatment (10 μM) for 5 days, **(B)** Molecular structure of CAPE. **(C)** IC50 of CAPE on AVICs, concentrations were transferred to Log(c); n = 5. **(D)** Cell morphology of AVICs with or without CAPE treatment (10 μM) for 5 days. **(E)** Alizarin Red S staining of the cells with different conditioned coloring: control (normal culture medium), OM (osteogenic medium), OM+CAPE (osteoblastic medium plus CAPE treatment); ^#^ *p* < 0.05 and ****p < 0.01 were accepted as significant difference, n=3.

To assess the toxic effects of CAPE on AVICs, we determined the half-maximal inhibitory concentration (IC50). CAPE was found to exhibit overt signs of toxicity when the concentration in the culture medium exceeded 10 μM (**Figure 1C**). Therefore, 10 μM CAPE was used for further experiments. The viability of AVICs cultured in the presence of CAPE was then evaluated in CCK-8 assays (**Figure 1B**: molecular structure). As shown in **Figure 1A**, compared with the control group, the viability of cells cultured in the presence of CAPE declined on day 5; however, no cytotoxicity was observed, even after 21 days of treatment (**Supplementary Figure 1**). Furthermore, there was no visible difference in the morphology of AVICs cultured with or without 10 μM CAPE for 5 days (**Figure 1D**).

### CAPE Inhibits OM-Induced Osteogenic Differentiation of AVICs

**Figure 2:**
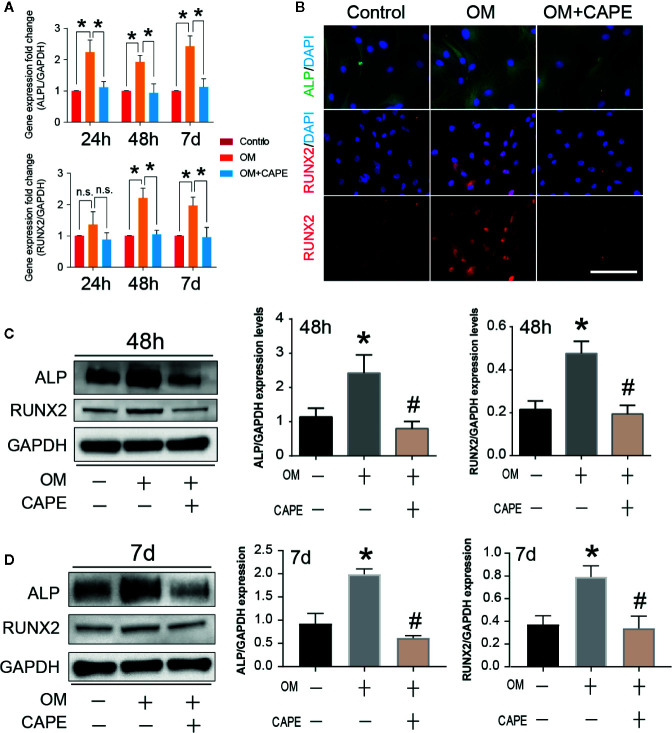
Effect of CAPE on OM induced calcific-related gene/protein expression in AVICs. **(A)** AVICs were stimulated with OM and then treated or not treated with 10 μM CAPE for 24 h, 48 h and 7 days, the mRNA expression levels of RUNX2, ALP were detected by qRT-PCR. **(B)** The immunofluorescent staining of ALP, RUNX2 on AVICs with above conditioned culturing for 48 h, **(C, D)** The protein expression levels of the above genes were determined by Western blot and quantification analysis for 48 h **(C)** and 7 days **(D)** treatment with CAPE. *p<0.05 compared with control, #*p*< 0.05 compared with OM.

Compared with the control group, significantly more AVICs were positively stained with Alizarin Red S staining after culture in OM for 21 days (* *P* < 0.05; **Figure 1E**). CAPE treatment resulted in a gradual decrease in Alizarin Red S positive staining compared with that of the OM group (* *P* < 0.05; **Figure 1E**) (**Supplementary Figure 2**). Subsequently, we analyzed the expression of the osteogenic differentiation-related genes RUNX2 and ALP in AVICs cultured in OM with or without CAPE for 24 h, 48 h, and 7 days (**Figure 2A**). Compared with the control group, OM significantly upregulated the expression of ALP and RUNX2 (* *P* < 0.05). With the addition of CAPE to the OM culture medium, ALP and RUNX2 were both significantly downregulated (# *P* < 0.05). Immunofluorescent staining of AVICs cultured in OM with and without CAPE for 48 h, revealed a similar pattern of ALP and RUNX2 protein expression (**Figure 2B**). Furthermore, following treatment with OM and CAPE for 48 h (**Figure 2C**) and 7 days (**Figure 2D**), the expression of RUNX2 and ALP at the protein level was significantly increased (* *P* < 0.05) compared with those detected in the control group (without OM and CAPE treatment), while the expression of these proteins was decreased compared to the levels detected in AVICs cultured in OM alone (# *P* < 0.05).

### Identification of DEGs and KEGG Pathway Analysis

**Figure 3:**
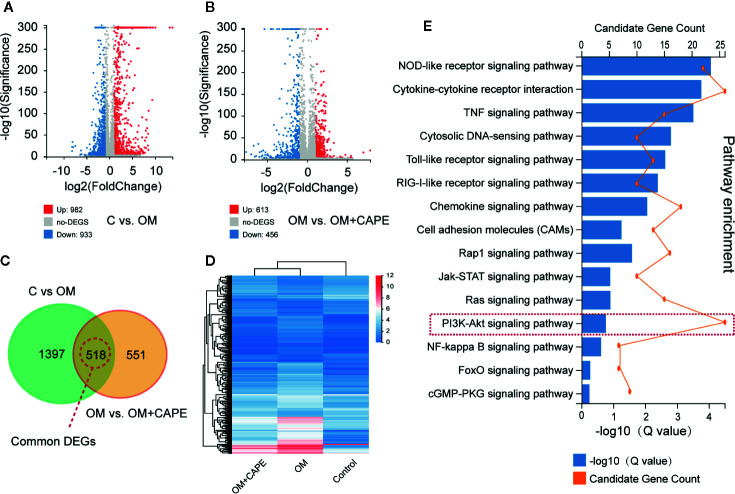
Gene expression profiles with RNA-sequencing under the OM (osteogenic medium) conditioned culturing with or without Caffeic Acid Phenethyl Ester (CAPE). **(A, B)** Volcano map of differentially expressed genes (DEGs) in C versus OM (log2(OM/C); **(A)**, up-regulation: 982 and down-regulation: 933); and OM versus OM + CAPE (log2(OM + CAPE/OM); **(B)**, up-regulation: 613 and down-regulation: 456), FC (fold change) > 1 was accepted as positive DEGs, **(C)** Venn interaction of DEGs of C versus OM (log2(OM/C) and OM versus OM+CAPE (log2(OM+CAPE/OM), **(D)** Heatmap for common DEGs gene expression with group clusters, **(E)** KEGG pathway enrichment, orange dots indicate the degree of enrichment (Q value (-Log10)), histogram indicates gene counts matched the pathway enrichment.

Compared with the control group, we observed marked differential gene expression (982 upregulated and 933 downregulated) in AVICs cultured in OM (**Figure 3A**). Furthermore, we observed marked differences in the global gene expression profiles of AVICs cultured in OM with and without CAPE (**Figure 3B**), with 1,069 DEGs (613 upregulated and 456 downregulated) detected in the presence of CAPE. Based on Venn diagrams of DEGs identified by comparison of the gene expression profiles in the OM versus control groups and the OM plus CAPE versus OM groups, we identified 518 common DEGs for further analysis (**Figures 3C, D**). KEGG signaling pathway enrichment analysis showed that these DEGs were highly enriched in functions related to the NOD-like receptor, TNF, PI3K-AKT, mTOR, NF-κB, and Toll-like receptor signaling pathways (**Figure 3E**).

### CAPE Inhibits Calcification of AVICs by Inhibiting NF-κB Activation

**Figure 4:**
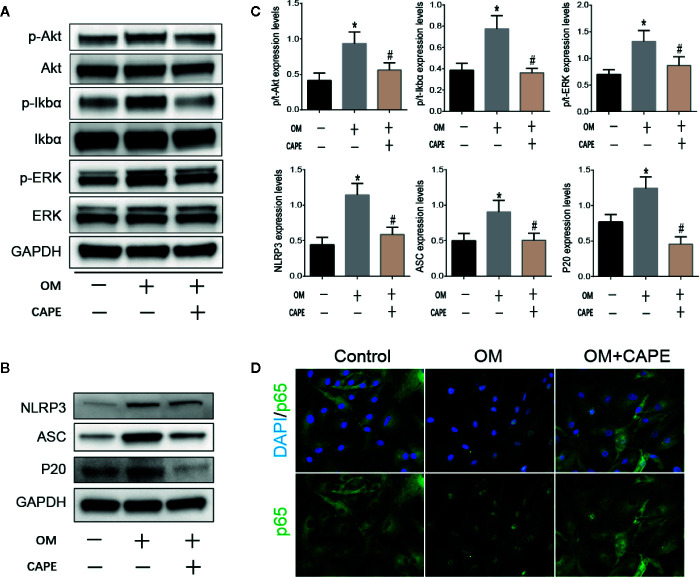
CAPE inhibits calcification of AVICs *via* interfering AKT, ERK1/2, NF-κB, and NLRP3 inflammasome activation. **(A)** Protein expression profiles of phosphorylated ERK, IκBα and AKT under the OM (osteogenic medium) conditioned culturing with or without CAPE, **(B)** The protein expression levels of NLRP3、 ASC、 P20 were determined by Western blot **(C)** Statistical analysis of p/t-ERK (phospho-Erk/total ERK), p/t-IkBa (phospho-IκBα/total-IκBα), p/t-AKT (phospho-AKT/total-AKT), and NLRP3, ASC, P20 were collected according to the gray semi-quantification. **(D)** Immunofluorescent staining for verifying that CAPE represses OM induced cell nucleus translocation of P65. *p < 0.05 compared with control and ^#^ *p* < 0.05 compared with OM showed significant difference.

Based on the results of RNA-seq analysis, we selected the NF-κB and PI3K-AKT pathway signaling for further studies. Compared with the control group, the protein levels of phospho-Erk, phospho-IκBα, and phospho-AKT were markedly increased in the OM group, and CAPE treatment decreased their expression, although the total levels of these proteins were unaffected (**Figures 4A, C**). In addition, CAPE inhibited nuclear translocation of NF-κB p65 in AVICs (**Figure 4D**). These findings indicated that activation of AKT, ERK1/2, and NF-κB was restrained in AVICs by the addition of CAPE to OM.

### CAPE Suppresses NLRP3 Inflammasome Activation in AVICs

The NLRP3 inflammasome is a novel target that regulates cell differentiation and inflammation (Sun et al., 2017). NF-κB is well-known to be a prerequisite for NLRP3 inflammasome activation (Afonina et al., 2017). Western blot analysis showed that protein expression levels of NLRP3, ASC, cleaved caspase-1 (P20) in AVICs were markedly increased in AVICs cultured in OM for 3 days, and that this effect was inhibited in the presence of CAPE (**Figures 4B, C**).

## Discussion

Many studies support the concept that CAVD is an active process involving multiple mechanisms, including abnormal calcium or phosphate metabolism, valvular inflammation, and pro-osteogenic reprogramming of AVICs (Aikawa and Libby, 2017). Our previous studies showed that many natural compounds with anti-inflammatory properties significantly inhibit valve calcification (Huang et al., 2020; Zhou et al., 2020). In this study, for the first time, we demonstrate that CAPE functions as an efficient inflammation inhibitor to suppress OM-induced calcification of human AVICs. Thus, our findings confirm the potential of anti-inflammatory interventions against CAVD.

In the current study, we first determined that 10 μM CAPE had no significant cytotoxic effects on AVICs but slowed cell proliferation over time. It has been widely reported that CAPE inhibits cell proliferation (Chang et al., 2017), and proliferation of AVICs has been linked with development of aortic valve calcification (Paradis et al., 2014). Thus, it is possible that CAPE prevents aortic valve calcification by suppressing cell growth.

Previous studies showed that AVICs from calcified aortic valves produce higher levels of pro-osteogenic biomarkers, including Runx2 and ALP (Rutkovskiy et al., 2017). In the present study, we demonstrated that OM induced increased expression of Runx2 and ALP, an effect that was inhibited by CAPE. To investigate the mechanism by which CAPE inhibited OM-induced calcification of AVICs, we performed a high-throughput gene expression analysis to rapidly and accurately identify the relevant molecular signaling pathways. DEGs selected by transcriptome sequencing were highly enriched in the TNF, PI3K-AKT, mTOR, NF-κB, Toll-like receptor, and NOD-like receptor signaling pathways. Of these, the NF-κB and NOD-like receptor signaling pathways are the most common inflammatory response-mediated signaling pathways.

The NLRP3 inflammasome, which is the core factor in NOD-like receptor signaling pathway, is a cytosolic complex involved in early inflammatory responses. It has been demonstrated that the NLRP3 inflammasome contributes to vascular smooth muscle cell phenotype switching, proliferation, and vascular remodeling in hypertension (Sun et al., 2017). NF-κB is a necessary prerequisite for NLRP3 inflammasome activation (Boaru et al., 2015). Following activation, NLRP3 forms a complex with its adaptor ASC, which facilitates the conversion of pro-caspase-1 to the active caspase-1 p10/p20 tetramer, leading to maturation of proinflammatory cytokines, such as IL-1β and IL-18 (F et al., 2002). In this study, we found that CAPE inhibited nuclear translocation of NF-κB p65 in AVICs and decreased the phosphorylated levels of IκBα. These results confirm that CAPE has a significant inhibitory effect on NF-κB activation. Furthermore, Western blot analysis of the protein expression of NLRP3, ASC, and P20 protein in AVICs cultured in OM in the presence of CAPE confirmed that CAPE effectively inhibited the activation of NLRP3, ASC, P20. These results indicate that the anti-calcification effect of CAPE depends on inhibition of the NF-κB/NLRP3 pathway. Moreover, it was shown that CAPE treatment markedly impaired the phosphorylation of AKT and ERK required to promote cell proliferation (Wang et al., 2019; Xu et al., 2019b). Thus, our findings confirm that mechanistically, CAPE inhibits the growth of AVICs by inhibiting the phosphorylation of AKT and ERK.

Therefore, CAPE reverses osteoblastic differentiation of aortic valve interstitial cells by regulating cell proliferation, inhibiting inflammation *via* AKT, ERK, NF-κB/NLRP3 pathways. Thus, our findings provide important clarification of the mechanism underlying the anti-calcification effects of CAPE.

## Conclusion

Our results suggest that CAPE significantly inhibits OM-induced calcification and phenotypic transformation of AVICs *via* signaling pathways including PI3K-AKT, ERK1/2, and NF-κB/NLRP3 inflammasome. Thus, CAPE represents a potential medical supplement to prevent the occurrence of CAVD.

## Data Availability Statement

The datasets analyzed in this article have been deposited in the Sequence Read Archive (SRA) database of NCBI under accession code PRJNA643215.

## Ethics Statement

The studies involving human participants were reviewed and approved by Ethics committee of Union Hospital, Tongji Medical College, Huazhong University of Science and Technology. The patients/participants provided their written informed consent to participate in this study.

## Author Contributions

KX, ML, and YH designed the project, collected the data, and wrote the manuscript. KX, TZ, YH, and FL analyzed the data, wrote and revised the manuscript. SC and GL revised the manuscript. ND and JS designed the project, gave financial support, and wrote the manuscript, and KX revised the manuscript. All authors contributed to the article and approved the submitted version.

## Funding

This work was supported by the National Key R&D Program of China (2016YFA0101100); the National Natural Science Foundation of China (81770387, 30371414, 30571839, 81700339, 81974034, 81670351; the Hubei Provincial Natural Science Foundation of China (2017CFB647); and the China Postdoctoral Science Foundation (Grant Number: 2018M630867).

## Conflict of Interest

The authors declare that the research was conducted in the absence of any commercial or financial relationships that could be construed as a potential conflict of interest.

## Supplementary Material

Supplementary Figure 1 Cell viability of CAPE treatment for 21 days.

Supplementary Figure 2 Two more repeats of **Figure 1E**, Alizarin Red S staining of the cells with different conditioned culuring: control (normal culture medium), OM (osteogenic medium), OM+CAPE (osteoblastic medium plus CAPE treatment).

Supplementary Table 1 List of qPCR primers.
