# Supplementary figures and images for: Caffeic Acid Phenethyl Ester Ameliorates Calcification by Inhibiting Activation of the AKT/NF-κB/NLRP3 Inflammasome Pathway in Human Aortic Valve Interstitial Cells
Source: Front Pharmacol. 2020 Jul 7;11:826. doi: 10.3389/fphar.2020.00826 (PMC7358518; doi:10.3389/fphar.2020.00826)

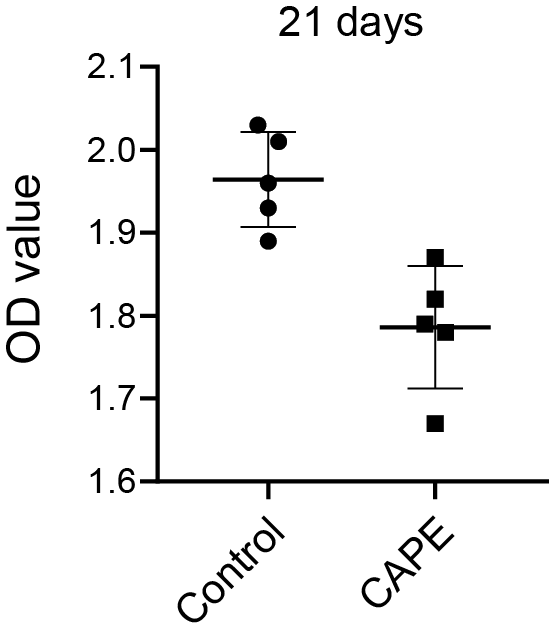

Supplement: Supplementary Figure 1 — Cell viability of CAPE treatment for 21 days. [file Image_1.tif]

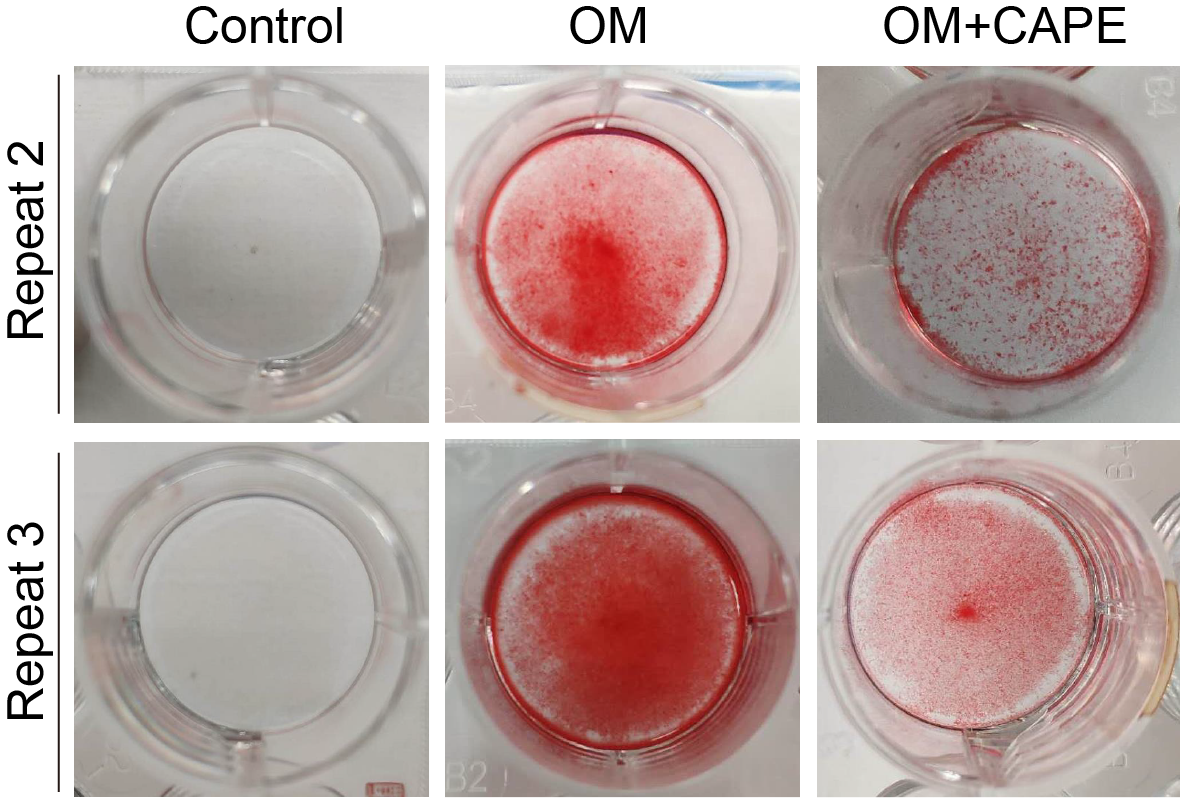

Supplement: Supplementary Figure 2 — Two more repeats of Figure 1E , Alizarin Red S staining of the cells with different conditioned culuring: control (normal culture medium), OM (osteogenic medium), OM+CAPE (osteoblastic medium plus CAPE treatment). [file Image_2.tif]
