# Supplementary material for: Caffeic Acid Phenethyl Ester Ameliorates Calcification by Inhibiting Activation of the AKT/NF-κB/NLRP3 Inflammasome Pathway in Human Aortic Valve Interstitial Cells
Source: Front Pharmacol. 2020 Jul 7;11:826. doi: 10.3389/fphar.2020.00826 (PMC7358518; doi:10.3389/fphar.2020.00826)
Supplement: Supplementary Table 1 — List of qPCR primers. [file Table_1.docx]

**Supplementary table 1. List of qPCR primers**

| **Gene symbol** | **Accession number** | **Primer sequence (3'-5')** | **Size (bp)** |
| --- | --- | --- | --- |
| RUNX2 | [NM_001024630.3](https://www.ncbi.nlm.nih.gov/entrez/viewer.fcgi?db=nucleotide&id=226442782) | CCGCCTCAGTGATTTAGGGC | 132 |
|  |  | GGGTCTGTAATCTGACTCTGTCC |  |
| ALPL | NM_000478.6 | CCGCTATCCTGGCTCCGTG | 136 |
|  |  | AGAGATGCAATCGACGTGGG |  |
| GAPDH | NM_002046.6 | ATGCCTCCTGCACCACCAACT | 218 |
|  |  | GATGACCTTGCCCACAGCCTTG |  |
